# Supplementary material for: Diagnostic Accuracy of Ex Vivo Confocal Microscopy for Surgical Margin Assessment of High-Risk Nodular Basal Cell Carcinoma
Source: Cancers (Basel). 2025 Sep 16;17(18):3019. doi: 10.3390/cancers17183019 (PMC12468694; doi:10.3390/cancers17183019)
Supplement: Supplementary file 1 [file cancers-17-03019-s001.zip › cancers-3807908-supplementary.pdf]

Supplementary Table S1. Characteristics of nodular, high-risk BCCs included in the study.

Tumor Location	Tumor Size (mm)	Recurrent
nose/peri-nasal	6	no
scalp	45	no
scalp	20	no
peri-oral	20	no
ear/peri-auricular	12	no
peri-oral	11	no
temple/lateral forehead	12	no
temple/lateral forehead	6	no
nose/peri-nasal	6	no
eyelid/peri-ocular	6	no
nose/peri-nasal	9	no
nose/peri-nasal	10	no
mandible/lower cheek	15	no
temple/lateral forehead	12	no
ear/peri-auricular	20	no
ear/peri-auricular	6	no
temple/lateral forehead	6	yes
nose/peri-nasal	6	no
nose/peri-nasal	10	no
ear/peri-auricular	6	no
nose/peri-nasal	8	no
nose/peri-nasal	5	no
temple/lateral forehead	7	no
temple/lateral forehead	12	no
peri-oral	11	no
nose/peri-nasal	10	no
mandible/lower cheek	7	no
nose/peri-nasal	7	no
nose/peri-nasal	9	no
nose/peri-nasal	5	no
nose/peri-nasal	8	no
temple/lateral forehead	25	no
ear/peri-auricular	11	no
temple/lateral forehead	6	no
temple/lateral forehead	6	no
nose/peri-nasal	8	no
nose/peri-nasal	5	no
nose/peri-nasal	13	no
temple/lateral forehead	7	no
temple/lateral forehead	5	no
nose/peri-nasal	12	no
ear/peri-auricular	12	no
ear/peri-auricular	10	no
peri-oral	12	no

temple/lateral forehead	8	no
nose/peri-nasal	10	no
nose/peri-nasal	6	no
temple/lateral forehead	10	no
temple/lateral forehead	7	no
peri-oral	10	no
temple/lateral forehead	6	no
